# AI Nurses Network: The Importance of Clinical Research Networks in Nursing

**DOI:** 10.1111/jnu.70042

**Published:** 2025-08-01

**Authors:** Siobhan O'Connor, Crina Grosan, Rebecca J. Oakey, Mengying Zhang, Xiaoyang Li, Emma Stanmore, David Woodcock, Jo Armes, Joanne Cull

**Affiliations:** ^1^ Florence Nightingale Faculty of Nursing, Midwifery and Palliative Care King's College London London UK; ^2^ Faculty of Life Sciences and Medicine King's College London London UK; ^3^ School of Health Sciences The University of Manchester Manchester UK; ^4^ Patient and Public Engagement and Involvement (PPIE) Representative Leeds UK; ^5^ School of Health Sciences University of Surrey Guildford UK; ^6^ Guy's and St Thomas' NHS Foundation Trust London UK; ^7^ London South Bank University London UK

**Keywords:** artificial intelligence, clinical research network, machine learning, natural language processing, nursing

1

Clinical research networks (CRNs) are growing in popularity due to the complex and interdisciplinary nature of health research and the need to generate robust scientific evidence that improves patient outcomes and healthcare delivery (Brown et al. 2016). These networks can include a range of nursing and other researchers, practicing clinicians from different specialties, Patient and Public Involvement and Engagement (PPIE) members, and administrative staff. Such networks can facilitate research collaboration in a topic area, strengthen education and training, and build researcher capacity alongside improving the research culture and environment (Robinson et al. 2015). Several nursing‐specific CRNs are active, such as a kidney care research network in the United Kingdom (Anderson 2020), a global network on disability nursing (Fisher et al. 2024), and a national clinical nurse leadership network in the United States (Bender et al. 2019), all of which report positive outcomes as well as challenges with setting up, running, and evaluating the networks activities. Nurses are also involved in broader multidisciplinary research networks that focus on specific research methods such as clinical trials (Gurwitz et al. 2022; Toups et al. 2023) or health‐related topics like COVID‐19 (DeVoe et al. 2020).

CRNs are often established to facilitate the generation of scientific evidence in a subject area and improve the flow of new knowledge, while supporting the professional development and career pipeline of clinical researchers. Ultimately, their goal is to improve healthcare delivery and quality of care by speeding up the translation of research evidence into professional practice, so new solutions to improve patient care and population health can be introduced into health systems nationally and internationally (Lopes et al. 2024).

With the emergence of artificial intelligence (AI) and its rapidly evolving pace, AI models and AI tools are starting to be developed and introduced in healthcare. A recent systematic review found many applications of AI in nursing across a broad range of areas of clinical practice (O'Connor et al. 2023). However, it highlighted that nurses lack of knowledge and skill in AI could hamper its development and integration in healthcare, as well as limit opportunities for nurses in their professional careers and their ability to support patients and families with AI‐based health technologies. A recent special issue of the *Journal of Nursing Scholarship* also focused on the role of AI in nursing and included a range of primary research studies, some of which emphasised the same barriers in relation to introducing AI in nursing (Sigma Nursing 2025).

With this in mind, a group of nursing scholars with support from colleagues in AI and data science established the world's first clinical research network on AI in nursing. After securing funding for the new CRN, we launched the AI Nurses Network (www.ai‐nurses.com) in December 2024 at the annual A Centre of Research for Nurses and Midwives (ACORN) Research Showcase at Guy's and St Thomas' NHS Foundation Trust in London. Drawing on lessons learned from previous and existing CRNs in nursing, we designed the network to support and advance the role of nurses in the field of AI by providing the profession with a suite of resources, events, and opportunities they can access and use for free. At present, the network has more than 900 members worldwide with nurses from the United Kingdom to Canada and Australia signed up. We also established an independent advisory board to offer ongoing advice and support to the new network.

To address the AI literacy gap in the nursing profession, we are utilising an existing online training program called “Innovation Scholars Programme: Enabling the big data revolution through skills training” funded by UKRI and sharing weblinks to the suite of free e‐learning courses on the AI Nurses Network website. The courses which cover a range of AI and data science topics are specifically tailored for the healthcare workforce to help professionals such as nurses learn the fundamentals of this new technological trend (Table 1). The knowledge and skills provided by these courses is aligned with NHS England's Artificial Intelligence and Digital Healthcare Technologies Capability Framework (NHS England 2025), enabling nurses to build up a range of digital competencies for their professional careers. An underpinning introductory course for those with no experience of what data is, as a precursor to the fundamentals in Table 1, is also available to access for free on Future Learn (https://www.futurelearn.com/courses/introduction‐to‐data‐science‐for‐healthcare‐professionals). In addition, we recorded an introductory webinar covering the key concepts in AI and their applications in nursing which is available on the AI Nurses Network dedicated YouTube channel (https://www.youtube.com/channel/UCavb447TmIdCICmik0nC9yQ). The channel also hosts regular webinars that we record with nurses around the world conducting research on AI in education and clinical practice to help spread knowledge on AI among the profession.

**TABLE 1:** AI and data science training courses from the Innovation Scholars Programme.

Pillar 1—Health data science	Pillar 2—Genomics	Pillar 3—Artificial intelligence
Introduction to Python for Health Research Artificial Intelligence in Health Analytics Machine Learning for Health Research Introduction to R for Health Research Predictive Modelling for Health Research Introduction to Statistics Natural Language Processing (Part 1)	Using Spreadsheets to Record Data and Metadata Basic R with Data Carpentry Statistics with R Advanced R (coming soon)	Demystifying AI Intermediate Python and Software Engineering Applied Artificial Intelligence for Health Research Ethics of AI (coming soon)

We are also delivering a series of bespoke online training workshops, running over the summer of 2025 and beyond, with some recorded and hosted on the networks You Tube channel. These are educating nurses on how to access and clean a digital health dataset to prepare it for predictive modeling, how to select appropriate machine learning algorithms or other AI techniques, and how to develop and validate an AI model for healthcare (Charow et al. 2021). Furthermore, we recently announced a student scholarship to support an undergraduate nursing student to get involved in the AI Nurses Network. The inaugural winner, a final year undergraduate children's nursing student, is taking part in AI research we are conducting (Moldovan et al. 2025; Rodger and O'Connor 2025). We hope the training resources and scholarships available will help equip nurses globally with essential AI expertise to help them generate evidence that informs professional practice and patient care.

The AI Nurses Network also offers seed funding to support the development of innovative and translational nurse‐led clinical research on AI, which creates positive change and value for patient care and population health (Morassaei et al. 2023). In particular, we sought to fund pilot studies that could benefit from financial support to transform into competitive applications for larger research funding. Our first seed funding call opened in early January and closed at the end of February 2025, offering two tiers of funding ranging from £10,000 to £20,000. We received numerous applications, which were rigorously evaluated, resulting in three funded research projects. The studies led by nurses from universities and NHS Trusts in England and Scotland cover a range of topics from cardiovascular health to postoperative recovery and nursing workflows and operational efficiency. We are looking forward to seeing the outcomes and impact of these nurse‐led AI research projects in the coming months and hope to be able to offer seed funding to nurses across the United Kingdom and beyond on an annual basis.

Finally, we promote various AI events and resources to nurses to ensure they can meet AI researchers, industry, and other stakeholders to launch their ideas for AI research as well as stay up to date with the latest cutting‐edge AI research, policies, and regulation, and other training and funding available for AI research. We set up and run a LinkedIn group dedicated to the AI Nurses Network (https://www.linkedin.com/groups/13072130/) and send out a monthly electronic newsletter to share AI‐related content with network members. The newsletters are also archived on the website and shared on the networks LinkedIn group. The newsletter summarises AI training, funding, and published research relevant to nursing and highlights upcoming AI conferences and other events that nurses can attend or access online. We are also planning our own series of events, starting with a hackathon on AI in nursing that will take place in London in September 2025. Hackathons in nursing have the potential to generate novel ideas along with start‐up projects and commercial ventures (Kagan et al. 2023) which we hope will benefit nurses who take part.

Although the AI Nurses Network is still in its infancy, there are early indications that it will help share AI knowledge among the profession, upskill nurses in AI, and facilitate nurse‐led AI research that improves patient care. From an AI perspective, the development of a CRN focused on AI in nursing is a timely and important step in supporting innovation in healthcare as AI has the potential to support nurses in multiple ways. However, for AI tools to be useful and safe in professional practice, they must be developed with input from healthcare professionals, especially nurses who work closely with patients and families. Nurses bring essential knowledge about patient care, clinical settings, and ethical concerns that can guide the development and deployment of AI tools that are practical, trustworthy, and effective (O'Connor et al. 2023). Hence, the AI‐Nurses Network is creating a space where nurses and AI experts can work together to design, implement, and evaluate AI‐based health technologies. Involving nurses early in the development of AI tools also helps build trust, improve skills, and encourage greater use of these technologies in everyday health and care.

As we move forward, we plan to expand the network internationally, secure further funding to enable an annual seed funding call and yearly scholarships, and establish new collaborations with nursing associations, researchers, clinicians, and policy makers. This will enable our CRN to build capacity for AI research in nursing and contribute to the pipeline of nursing scholars who pioneer AI models and AI tools that improve patient care and population health.

## Disclosure

Clinical resources: The AI Nurses Network and its resources can be found online: https://www.ai‐nurses.com/.

## Conflicts of Interest

Professor Emma Stanmore is the Director of KOKU Health Ltd., a non‐profit company in the UK (https://kokuhealth.com/). All the other authors have no competing interests to declare that are relevant to the content of this article.

## Data Availability

The authors have nothing to report.
